# Benralizumab and the integrated management of co‐morbid severe eosinophilic asthma with chronic rhinosinusitis with nasal polyps

**DOI:** 10.1002/clt2.70051

**Published:** 2025-04-08

**Authors:** Joaquim Mullol, Maria D'Amato, Eugenio de Corso, Joseph K. Han, Jody Tversky

**Affiliations:** ^1^ Rhinology Unit & Smell Clinic ENT Department Hospital Clínic Barcelona Clinical and Experimental Respiratory Immunoallergy (IDIBAPS) Universitat de Barcelona CIBER de Enfermedades Respiratorias (CIBERES) Barcelona Catalonia Spain; ^2^ Respiratory Department Monaldi Hospital‐AO Dei Colli‐Naples Naples Italy; ^3^ Otolaryngology Head and Neck Surgery Fondazione Policlinico Universitario A. Gemelli IRCSS Rome Italy; ^4^ Department of Otolaryngology Head and Neck Surgery Eastern Virginia Medical School Norfolk Virginia USA; ^5^ Division of Allergy & Clinical Immunology Johns Hopkins University School of Medicine Baltimore Maryland USA

**Keywords:** benralizumab, biologics, CRSwNP, eosinophilia, severe eosinophilic asthma

## Abstract

**Background:**

Type 2 (T2) inflammation, characterized by blood and airway eosinophilia, underlies severe eosinophilic asthma (SEA) and chronic rhinosinusitis with nasal polyps (CRSwNP). In line with the Global Airways theory, SEA and CRSwNP frequently co‐occur, creating a multimorbid phenotype. Separately, SEA and CRSwNP are burdensome: when concomitant, they compound each other, creating a more difficult‐to‐treat disease with increased complications.

**Body:**

Current management approaches rarely control disease and are associated with substantial side‐effects. Several recently developed anti‐IL‐5 monoclonal antibodies have shown efficacy in treating co‐morbid SEA with CRSwNP by targeting T2 inflammation with systemic therapies. Of these, only benralizumab directly targets the IL‐5 receptor‐α, leading to rapid, sustained, near‐complete eosinophil depletion. Analyses in patients with co‐morbid SEA with CRSwNP are limited, although data from the ANDHI, XALOC‐1, and RANS studies suggest benralizumab can effectively target inflammation underlying co‐morbid disease.

**Conclusion:**

Despite progress toward more effective therapies, treatment approaches remain siloed, with SEA and CRSwNP often managed separately. There is a need for the development of multidisciplinary approaches for treating patients with comorbid SEA with CRSwNP.

## 1 INTRODUCTION

The Global Airways theory posits that upper airway diseases, such as chronic rhinosinusitis with nasal polyps (CRSwNP), and lower airway diseases, such as severe eosinophilic asthma (SEA), while considered distinct clinical entities (phenotypes), converge (as endotypes) via shared underlying disease processes (a concept also known as Global Airways Disease).^1^, ^2^, ^3^ In the cases of SEA and CRSwNP, this common underlying process is type 2 (T2) inflammation.^1^, ^2^, ^3^, ^4^, ^5^

SEA itself is a subtype of asthma characterized by a high level of eosinophils and significant inflammation and airway obstruction, typically occurring in patients (often middle‐aged women with high sensitivity to oral corticosteroids) who experience persistent asthma symptoms despite receiving standard treatments including high‐dose inhaled corticosteroids.^6^, ^7^ Clinical trials have demonstrated markedly improved outcomes in both patients with SEA and those with severe CRSwNP, when treated with monoclonal antibody therapies that attenuate the T2 inflammatory response by targeting immunoglobulin‐E (IgE) and T2 cytokines, or their receptors.^1^, ^4^, ^5^, ^8^

In addition, recognition of the need for integrated management of health conditions across the medical spectrum is increasing.^2^, ^9^, ^10^, ^11^, ^12^, ^13^ Indeed, there have already been successes in the integrated management of patients with chronic obstructive pulmonary disease, asthma, and diabetes.^14^, ^15^, ^16^ Currently, SEA and CRSwNP are typically managed by specialists in different fields, impeding cohesive management of co‐morbid disease and adversely affecting patient outcomes.^1^, ^17^ An integrated, multidisciplinary, approach is urgently needed to address this issue, enhancing the overall quality of life for patients with co‐morbid SEA with CRSwNP and simultaneously limiting concomitant conditions.^2^, ^18^

This article will:

1. Describe the shared inflammatory mechanism underlying SEA and CRSwNP.
2. Assess evidence supporting the application of benralizumab in treating comorbid SEA with CRSwNP, largely based on real‐world data.
3. Discuss the need to move from a siloed healthcare approach to an integrated multidisciplinary treatment strategy.

## 2 METHODS

We performed a search of the literature over the last 10 years using PubMed and the following search terms in the Title/Abstract of articles: “benralizumab” AND “severe eosinophilic asthma” AND “chronic rhinosinusitis with nasal polyps” OR “CRSwNP.” We reviewed the titles, abstracts and reference lists of the search results and agreed on the most relevant studies for discussion in this narrative review. In general, studies were preferentially selected if they were recent and/or large prospective randomized trials with a robust analytical design.

### 2.1 Disease mechanism and burden

Chronic T2 inflammation, characterized by a long‐term increase in eosinophil numbers in the blood and airway tissue, underlies both SEA and CRSwNP.^4^, ^5^, ^19^, ^20^ The T2 inflammatory pathway produces a signaling cascade in response to factors including (but not limited to) allergens, viral infections, and air pollution, the reaction to which is mediated by the pro‐inflammatory alarmin cytokines thymic stromal lymphopoietin, interleukin (IL‐) 25 and 33, and the effector cytokines IL‐4, 5, and 13.^3^, ^5^, ^21^ In SEA, IgE sensitization also plays a crucial role; as allergens enter the body they trigger an immune response mediated by IgE antibodies which leads to the activation of mast cells and basophils and the recruitment and activation of eosinophils, further intensifying inflammation in the airways.^5^, ^22^, ^23^

This signaling cascade and IgE sensitization promote processes including epithelial disruption, goblet cell hyperplasia, increased vascularization and tissue hypertrophy and remodeling, in addition to release of inflammatory mediators such as histamine and leukotrienes which further exacerbate airway inflammation and hyperreactivity and ultimately result in the signs and symptoms of SEA and CRSwNP.^5^, ^22^, ^23^ Specifically, in the case of SEA, these include variable airflow limitation and mucus hypersecretion leading to wheezing, shortness of breath and chest tightness, and, in the case of CRSwNP, inflammation‐based nasal obstruction/congestion combined with anterior and/or posterior nasal discharge, which may be combined with a reduction in/loss of smell, and facial pain/pressure.^5^, ^23^, ^24^, ^25^

Given the shared underlying etiology and the implications of the Global Airways theory, it perhaps should not be surprising that 40%–70% of patients with CRSwNP have concomitant asthma, and 70%–90% of patients with severe asthma have concomitant CRSwNP.^1^, ^5^ Furthermore, 10% of patients with CRSwNP (up to 30% with severe CRSwNP) and 15% with severe asthma live with aspirin‐ or non‐steroidal anti‐inflammatory (NSAID)‐exacerbated respiratory disease (AERD/N‐ERD); a triad entailing T2 inflammation‐related SEA, CRSwNP, and respiratory reactions to cyclooxygenase inhibitors (e.g., aspirin and NSAIDs).^5^, ^26^, ^27^

Separately, SEA and CRSwNP can impose substantial burdens in terms of healthcare‐related quality of life (HRQoL) and healthcare resource use (HCRU).^4^, ^5^, ^28^, ^29^, ^30^ When they occur together in a patient they not only compound each other, but also complicate treatment.^1^, ^4^, ^5^, ^17^, ^31^ Additionally, the association of co‐morbid SEA with CRSwNP with intolerance to common, low side‐effect, analgesics and NSAIDs in AERD/N‐ERD further increases the severity of the disease and the difficulty of its treatment, exacerbating impacts on HRQoL. The current steroid‐dependent management approaches for these patients are associated with significant side‐effects, and even when combined with surgery to address CRSwNP specifically, physicians may struggle to reach effective disease control.^32^, ^33^, ^34^ Despite this, there has not yet been any large‐scale integrated program of research concerning the HRQoL and HCRU‐related burdens of this co‐morbid condition. As such, given the consequential risks and burdens faced by patients with co‐morbid SEA with CRSwNP, there is an urgent need for clinical focus on this condition.

### 2.2 Potential role of monoclonal antibodies in the management of co‐morbid SEA with CRSwNP

**FIGURE 1:**
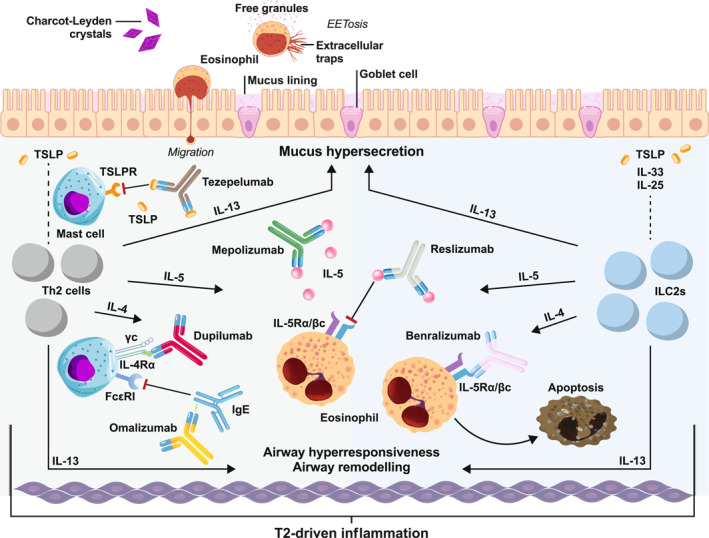
The pathophysiological mechanisms of T2 inflammation and monoclonal antibody‐based biologic drugs targeting these pathways. Figure adapted with permission from Jackson D. J. et al.^22^ **T2 inflammation:** Epithelial cytokines (IL‐33 and IL‐25 and TSLP) can activate ILC2 and type 2 Th2 to produce type 2 cytokines (IL‐4, IL‐5 and IL‐13). IL‐4 and IL‐13 promote B cell differentiation into IgE‐producing cells, which bind to receptors on mast cells and basophils and can trigger bronchoconstriction, mucus production, increased vascular permeability and edema. Furthermore, IL‐13 can induce goblet cell hyperplasia and mucus hypersecretion and encourage eosinophil recruitment and IL‐5 can also recruit and activate eosinophils. Once activated, eosinophils release ILC2‐promoting cytokines causing reciprocal upregulation of both cell types and associated type 2 immune responses. Thus, these type 2 cytokines drive activation of the airway epithelial cells and remodeling of the epithelium. Furthermore, transepithelial eosinophil migration followed by eosinophil ETosis and the formation of Charcot‐Leyden crystals contribute to airway obstruction. **Monoclonal antibody‐based biologic drugs:** Mepolizumab and resiluzmab bind to IL‐5, interfering with eosinophil signal transduction and thereby preventing eosinophil proliferation. Omalizumab binds directly to circulating IgE, preventing it from binding to FcεRI on mast cells. Benralizumab directly binds to IL‐5 receptors, leading to lymphcyte‐mediated apoptosis of eosinophils. Tezepelumab binds to TSLP, preventing it from binding to the TSLP‐receptor on mast cells. Dupilumab binds directly to the IL‐4Rα on mast cells and blocks the signaling of both IL‐4 and IL‐13. EET, eosinophil ETosis; FcεRI, high‐affinity IgE receptor; IgE, immunogloblin E; IL, interleukin; IL‐4Rα, interleukin‐4 receptor alpha; IL‐5Rα, interleukin‐5 receptor alpha; ILC2, group two innate lymphoid cell; Th, T helper; TSLP, thymic stromal lymphopoietin; TSLPR, thymic stromal lymphopoietin receptor; T2, type 2; βc, beta common chain receptor; γc, gamma common chain receptor.

Figure 1 provides an overview of the pathophysiological mechanisms of T2 inflammation and shows how recently developed monoclonal antibody‐based biological drugs, such as benralizumab, dupilumab, mepolizumab, omalizumab, reslizumab, and tezepelumab target various T2 pathways.^3^, ^4^, ^22^, ^35^

Based on the available clinical trial evidence in people with CRSwNP, omalizumab, mepolizumab and dupilumab are the only currently approved (and re‐imbursed) treatments worldwide for this condition.^24^, ^36^ Emerging clinical data on the efficacy of other biologic therapies in people with CRSwNP are eagerly anticipated.

Benralizumab, meplizumab and reslizumab are approved specifically for SEA and dupilumab and tezepelumab can also be used in the treatment of severe asthma (regardless of phenotype). Benralizumab is the only monoclonal antibody that binds directly to IL‐5 receptors, leading to potent natural‐killer (NK) lymphocyte‐mediated apoptosis of eosinophils (Figure 1).^3^, ^37^ Per the results of pivotal benralizumab phase 3 studies (SIROCCO [NCT01928771] and CALIMA [NCT01914757]), this leads to a rapid depletion of blood eosinophil counts to near‐zero levels, a no less dramatic reduction in inflammation, and significant improvements in outcomes for patients with SEA.^38^, ^39^

Consequently, a number of expert consensus statements now support the use of biologics such as benralizumab, for the treatment of SEA with or without co‐morbid CRSwNP.^2^, ^10^, ^12^, ^17^, ^40^

### 2.3 Benralizumab—Randomized clinical trials (RCTs)

While various randomized clinical trials have separately focused on the efficacy of benralizumab as a therapy for SEA^8^, ^38^, ^39^ or for CRSwNP,^41^, ^42^ none have yet focused on patients with co‐morbid SEA with CRSwNP.

**FIGURE 2:**
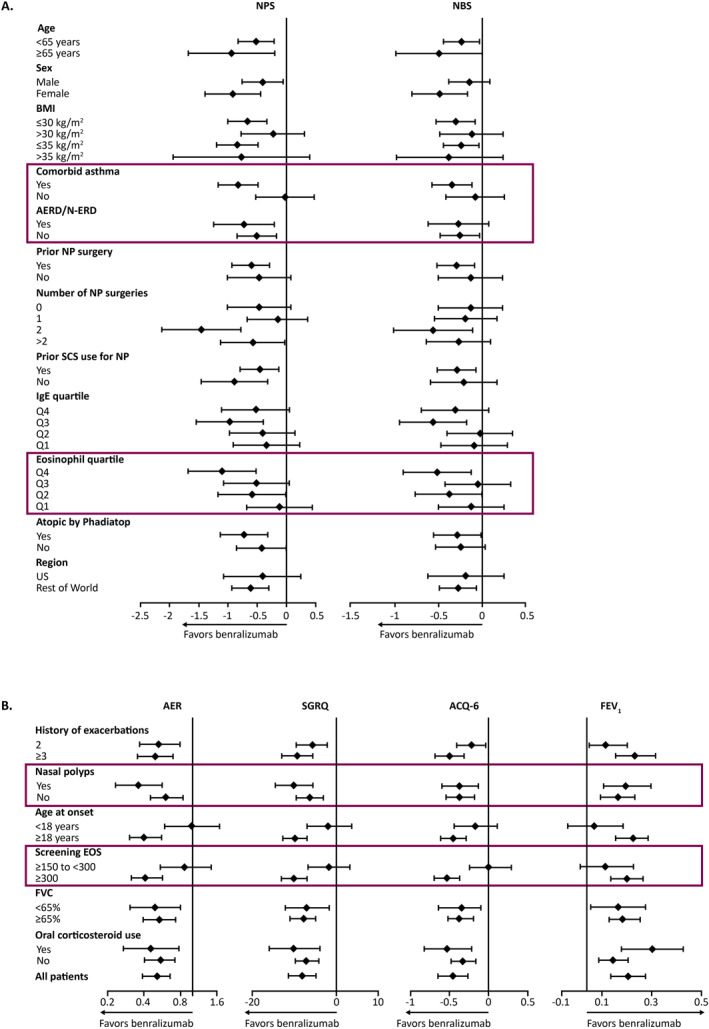
Changes in measures of SEA and NP severity in response to benralizumab therapy in the (A) OSTRO study (reprinted with permission.^41^ Copyright 2022, Elsevier) and (B) ANDHI study (reprinted with permission.^8^ Copyright 2021, Elsevier). ACQ‐6, six‐item Asthma Control Questionnaire; AER, annualized exacerbation rate; AERD, aspirin‐exacerbated respiratory disease; BMI, body mass index; EOS, eosinophil count at screening; FEV_1_, forced expiratory volume in one second; FVC, forced vital capacity; IgE, immunoglobulin E; NBS, nasal blockage score; N‐ERD, NSAID‐exacerbated respiratory disease; NP, nasal polyps; NPS, nasal polyp score; NSAID, non‐steroidal anti‐inflammatory drug; Q, quarter; SCS, systemic corticosteroids; SGRQ, St George's Respiratory Questionnaire; US, United States of America.

The phase 3 OSTRO (Efficacy and Safety of Benralizumab in Chronic Rhinosinusitis with Nasal Polyps; NCT03401229) study included patients with symptomatic severe CRSwNP despite treatment with intranasal corticosteroids who had a history of systemic corticosteroid use and/or surgery for nasal polyps. A high proportion of patients (∼70%) had co‐morbid mild asthma.^41^ Response to benralizumab (measured by the co‐primary endpoint of nasal blockage score and nasal polyp score) was found to be more pronounced in patients with co‐morbid mild asthma and higher baseline eosinophil counts (Figure 2). Additionally, following benralizumab treatment, these patients experienced substantial improvements in six‐item Asthma Control Questionnaire score (ACQ‐6; difference from placebo −0.330 [95% CI: −0.748, 0.089]; *p* = 0.12) and annualized asthma exacerbation rate (AER; rate ratio 0.53 [95% CI: 0.17, 1.63]; *p* = 0.16) (Table 1).^41^ It should be noted that as a CRSwNP study, OSTRO was not designed to assess the effect of treatment on asthma, and that patients in OSTRO with co‐morbid disease had mild (as opposed to severe) asthma.

**TABLE 1:** Summary of current evidence supporting the efficacy of benralizumab in patients with co‐morbid disease.

Study	Focus	Design	Outcomes in patients with co‐morbid disease
OSTRO (NCT03401229)^41^	CRSwNP	Phase 3, randomized, double‐blind, 56‐week, placebo‐controlled study evaluating the efficacy and safety of benralizumab versus placebo, and identifying demographic, laboratory, and clinical characteristics that predict response to benralizumab therapy in a population of patients with severe, symptomatic CRSwNP despite standard‐of‐care therapy (*N* = 413)	In patients with co‐morbid CRSwNP with mild asthma (*n* = 278): At week 40, a decrease from baseline in ACQ‐6 score was observed in patients taking benralizumab (−0.195) but not in those receiving placebo (0.135). Improvements in ACQ‐6 score favored benralizumab versus placebo at both week 40 (between‐group difference: −0.330 [95% CI: −0.748, 0.089]; *p* = 0.12) and week 56 (−0.331 [95% CI: −0.795, 0.133]; *p* = 0.16) AER was 47% lower with benralizumab than with placebo at week 40 (0.06 and 0.12 events per year, respectively; rate ratio: 0.53 [95% CI: 0.17, 1.63])
ANDHI (NCT03170271)^8^, ^43^	SEA	Phase 3b, randomized, double‐blind, parallel‐group, 24‐week, placebo‐controlled trial designed to investigate the efficacy of benralizumab in addition to standard‐of‐care asthma therapy for patients with uncontrolled SEA (*N* = 153; CRSwNP sub‐study)	In patients with co‐morbid SEA with CRSwNP: Patients receiving benralizumab had a greater improvement in SNOT‐22 scores between baseline and week 24, compared with patients taking placebo (RR: −8.91 [95% CI: −16.42, −1.40]; *p* = 0.0204) Patients receiving benralizumab had a significantly greater least squares mean change from baseline in SNOT‐22 scores, compared with those receiving placebo, from the first time point assessed, increasing over the course of the study (mean difference in change from baseline at week 4: −9.07 [*p* = 0.0076]; week 12: −11.63 [*p* = 0.0034]; and week 24: −10.44 [*p* = 0.0176]) A higher percentage of patients receiving benralizumab achieved clinically meaningful improvements from baseline in SNOT‐22 scores at week 24, compared with those receiving placebo (71.3% vs. 45.5%; OR: 2.99 [95% CI: 1.43, 6.24]; *p* = 0.0036). The magnitude of the effect was further enhanced for patients with high baseline SNOT‐22 scores (79.7% vs. 48.8%; OR: 4.11 [95% CI: 1.80, 9.39]; *p* = 0.0008)
RANS (NCT05180357)^44^	Co‐morbid SEA and CRSwNP	Retrospective, 12‐month, study using medical chart reviews of adult patients with SEA and concomitant CRSwNP from the US, Italy, France, Spain, and Japan who initiated treatment with benralizumab (*N* = 233)	The following changes were observed between baseline and 12 months in patients receiving benralizumab: A decrease in mean (SD) SNOT‐22 score of −19.8 (95% CI; −23.6, −15.9), from 47.5 (22.6) to 28.9 (21.1) A decrease in mean (SD) NPS of −1.2 (95% CI; −1.7, −0.6), from 3.8 (2.4) to 3.0 (2.1) A decrease in mean (SD) ACQ‐6 score of 0.8 (95% CI; −1.1, −0.4), from 1.6 (1.3) to 0.8 (1.0) A decrease in AER from 1.2 (95% CI: 1.0, 1.4) to 0.2 (95% CI: 0.1, 0.3) During the 12‐month post‐index period, 67.6%, 49.1%, and 56.6% of patients taking benralizumab achieved clinically meaningful improvements in the SNOT‐22 total score, total NPS, and ACQ‐6 score, respectively
MEGA^45^	Co‐morbid SEA with CRSwNP	Multicenter, noninterventional, retrospective, observational, real‐life study in the allergology and pulmonology departments of nine hospitals belonging to the Spanish asthma network (*N* = 206)	A total or partial improvement in loss of smell was found in 39.1% of patients after treatment with benralizumab; partial improvement in 17.0%, total improvement in 22.0%. Olfaction was more likely to improve in patients with atopy, short systemic corticosteroid cycles, and greater nasal polyp size Patients who experienced total improvement in olfaction had a lower frequency of asthma exacerbations, versus those with no improvement (*p* = 0.008)
XALOC‐1 (ANANKE, NCT04272463; BETREAT, N/A; BPAP, N/A; ORBE II: NCT04648839, VOLTS, N/A)^46^, ^47^, ^48^, ^49^	SEA	Multinational, retrospective, observational, real‐world, 24‐month, program composed of five national studies investigating the use of benralizumab in patients with SEA for up to 96 weeks in Canada (VOLTS), Italy (ANANKE), Portugal (BETREAT), Spain (ORBE II), and the UK (BPAP) (overall, *N* = 316; ORBE II, *N* = 75; ANANKE, *N* = 205)	*Exacerbations*: Benralizumab substantially reduced AER in both groups by week 48, from 3.77 to 0.16 in patients with co‐morbid SEA with CRSwNP (reduction, −95.8%) and from 4.37 to 0.37 in patients with SEA alone (−91.5%). For severe exacerbations, benralizumab reduced AER from 1.06 to 0.01 in patients with co‐morbid SEA with CRSwNP (−99.1%) and from 1.16 to 0.09 in patients with SEA alone (−92.2%). By week 48, 89.5% of patients with co‐morbid SEA with CRSwNP and 71.9% of patients with SEA alone were exacerbation free. Overall, the presence of CRSwNP was associated with a lower risk of exacerbations (*p* = 0.0017) and severe exacerbations (*p* = 0.025) in patients treated with benralizumab *OCS*: Patients with co‐morbid SEA with CRSwNP were less likely to be OCS dependent after 1 year of follow up, compared with patients with SEA alone (6.9% vs. 14.8). Additionally, a higher percentage of patients with SEA with CRSwNP completely withdrew maintenance OCS compared with those with SEA alone (70.6% vs. 47.2%). Patients with co‐morbid SEA with CRSwNP reduced the median (Q1–Q3) daily dose of maintenance OCS from 10 (5.0–32.0) mg at baseline to 0.0 (0.0–5.0) at 1 year‐follow up, and had an 87.5% mean reduction in the daily OCS dose, with 58.3% of patients achieving a daily dose reduction of ≥50% In patients with co‐morbid SEA with CRSwNP, mean OCS dose (measured in prednisone‐equivalent milligrams) decreased from 15.7 ± 9.2 mg/daily at baseline to 5.6 ± 7.9 mg/daily by week 48; a reduction of 64.3%. In total, 50% of patients with co‐morbid SEA with CRSwNP were able to eliminate long‐term OCS and 57.1% achieved a reduction in OCS dose. In patients with SEA alone, mean OCS dose was reduced from a baseline of 10.3 ± 7.8 mg/daily to 6.9 ± 8.1 mg/daily at week 48 (−33%). 31.8% of patients with SEA alone completely discontinued OCS use and 37.5% accomplished a reduction in OCS dose *PROs*: By week 48, 73.0% of patients with co‐morbid SEA with CRSwNP and 65.2% of those with SEA alone experienced improvements matching or exceeding MCID in ACQ‐6 (−0.5) or ACT (3) scores *Lung Function*: Improvements were confirmed in both patient subgroups, although those in patients with co‐morbid SEA with CRSwNP were greater; mean (SD) increase in pre‐BD FEV_1_ of 426 (420) mL versus 277 (401) mL in patients with SEA alone. Patients with co‐morbid SEA with CRSwNP were also more likely than those with SEA alone to have a pre‐BD FEV_1_ ≥80% at 1‐year follow‐up (52.2% vs. 43.2%) *HCRU*: Patients with SEA with CRSwNP experienced a 97% reduction in the rate of hospitalizations and an 88% reduction in visits to the emergency department (vs. 89% and 85%, respectively, in patients with SEA alone)
N/A^50^	SEA	Multicenter, retrospective, observational study on patients with SEA treated with benralizumab who were referred to 12 specialized Italian secondary care facilities in the “southern Italy network on severe asthma therapy” (*N* = 137 for total study)	*PROs*: In patients with co‐morbid SEA with CRSwNP (*n* = 79), SNOT‐22 decreased from 46 at baseline to 32 at 24 weeks (*p* < 0.0001). At baseline, 38 (48.1%) of this patient group reported severe symptoms, 34 (43%) moderate symptoms and 7 (9%) mild symptoms. After 24 weeks, 15 (19%) from the severe group were reclassified as moderate, 2 (3%) as mild, and 4 (5%) went from moderate to mild. Seventy three (92%) patients reported ACT MCID and 30 (38%) patients SNOT‐22 MCID. Twenty‐nine patients (37%) reported both ACT and SNOT‐22 MCID. There were no statistically significant differences between patients with both upper and lower airway symptom improvement and those who improved only lower airway outcome measures, except for SNOT‐22, which was higher in patients who reported ACT and SNOT‐22 MCID (42 vs. 58, *p* = 0.0037). More co‐morbid SEA with CRSwNP patients versus SEA alone patients had ACT MCID (92 vs. 79%, *p* = 0.0387) *CRSwNP severity*: In patients with co‐morbid SEA with CRSwNP, there was a reduction from baseline of sinus opacification at 24 weeks in 16 patients who underwent sinus‐CT scans; the intracIass correlation co‐efficient between raters was 0.96 (0.92–0.99) for assessing the Lund Mackey score, which decreased from 12.4 (8.8–16.1) to 6.4 (3.5–9.4) (*p* = 0.02) *Lung function*: More co‐morbid SEA with CRSwNP patients showed enhanced responses at 24 weeks compared with SEA alone, for FEV_1_% (23.1 vs. 13%, *p* = 0.017) and FEF_25‐75%_ (22 vs. 11%, *p* = 0.0362) *OCS*: There was a numerical (but not statistical) difference between co‐morbid SEA with CRSwNP versus SEA alone groups for suspension of OCS (77 vs. 62%, *p* = 0.2175) and remaining exacerbation‐free despite OCS discontinuation (70 vs. 53%, *p* = 0.1617)
N/A^51^	SEA	Real‐life, multicentre, retrospective and observational study about the therapeutic effects of benralizumab on the upper and lower airways of patients with SEA over 2 years of treatment (*N* = 164)	At year 2 in patients with co‐morbid SEA with CRSwNP (*n* = 72): A persistent improvement in CRSwNP (defined as SNOT‐22 < 30 and no nasal polyp recurrence) was observed in 33 (40%) patients (at baseline, no patient satisfied both criteria) 45 patients (55%) had SNOT‐22 < 30 at year 2 (from 4 patients [5%] at baseline) 59 patients (72%) had no CRSwNP recurrence (from 17 patients [21%] at baseline 24 patients (29%) met the criteria for persistent improvement in CRSwNP and sustained clinical remission on‐treatment of SEA (no exacerbations, no OCS, pre‐bronchodilator FEV 1 ≥80% pred., ACT score ≥20)

Abbreviations: ACQ‐6, 6‐item Asthma Control Questionnaire; ACT, Asthma Control Test; AER, annualized exacerbation rate; BD, bronchodilation; CI, confidence interval; CRSwNP, chronic rhinosinusitis with nasal polyps; FEF_25‐75%_, Forced expiratory flow between 25% and 75% of forced vital capacity; FEV_1_, forced expiratory volume in one second; HCRU, healthcare resource use; MCID, minimally clinically important difference; N/A, not applicable; NP, nasal polyps; NPS, nasal polyps score; OCS, oral corticosteroids; OR, odds ratio; PROs, patient‐reported outcomes; RR, relative reduction; SD, standard deviation; SEA, severe eosinophilic asthma; SNOT‐22, sino‐nasal outcome test‐22.

The phase 3 ANDHI (Assessment of the Efficacy of Benralizumab in Patients with Severe Eosinophilic Asthma; NCT03170271) study included patients with SEA who had experienced ≥2 prior‐year exacerbations despite high‐dosage inhaled corticosteroid plus additional controller(s). ^8^, ^43^ Of the ANDHI population (*N* = 656), 34.8% reported having a medical history of nasal polyps of any severity at study entry and 23.3% had evidence of physician‐diagnosed ongoing nasal polyps at baseline and were included in the nasal polyp sub‐study. ^8^, ^43^ In the overall population, response to benralizumab in terms of asthma response (measured by ACQ‐6, AER, St George's Respiratory Questionnaire, and pre‐bronchodilator forced expiratory volume in one second [FEV_1_]), was found to be more pronounced in patients with concomitant CRSwNP or higher baseline eosinophil counts (Figure 2) with a more pronounced effect of benralizumab therapy in patients with co‐morbid CRSwNP compared with patients with co‐morbid CRS without nasal polyps.^8^ In the nasal polyps sub‐study of patients with co‐morbid SEA with CRSwNP, there was significant and rapid reductions in CRSwNP‐specific SNOT‐22 scores in those receiving benralizumab compared with placebo (relative reduction [RR]: −8.91 [95% CI: −16.42, −1.40]; *p* = 0.0204) (Table 1).^43^ Furthermore, patients with high baseline SNOT‐22 scores experienced significant reductions in those scores with benralizumab compared with placebo (RR: −10.44; *p* = 0.0176).^43^

### 2.4 Benralizumab—Real‐world evidence

We identified four real‐world studies^44^, ^45^, ^52^, ^53^ retrospectively looking at the efficacy of benralizumab in patients with co‐morbid SEA with CRSwNP. Only three of these studies were of any substantial scale (Retrospective, Observational Study in Patients With Severe Eosinophilic Asthma and Nasal Polyps Treated by FASENRA® [RANS; NCT05180357], MEchanism underlying the Genesis and evolution of Asthma [MEGA], and a study by Bajpai et al. conducted in 2021), and only two (the RANS and MEGA studies) of which considered the effects of benralizumab separately from those of other IL‐5 inhibitors (Table 1). Additionally, country‐level data from sub‐analyses of the XALOC‐1 real‐world study program (ANANKE, NCT04272463; BETREAT; BPAP; ORBE II, NCT04648839; VOLTS) and from analyses using data from specialized respiratory care facilities in Southern Italy, have provided some evidence regarding the efficacy of benralizumab as a therapy for co‐morbid SEA with CRSwNP^46^, ^47^, ^48^, ^49^, ^50^, ^51^, ^54^ (Table 1).

These real‐world studies showed similar incidental results to those seen in RCTs of benralizumab, including the pivotal SIROCCO and CALIMA phase 3 studies that led to approval of benralizumab in SEA.^38^, ^39^, ^54^ In RANS, for example, patients with SEA receiving benralizumab experienced statistically and clinically significant improvements in metrics of CRSwNP severity during the 12‐month post‐index period.^44^ Furthermore, while XALOC‐1 primarily examined the effect of benralizumab on multiple measures of SEA control (i.e. HCRU, airway obstruction, and patient‐reported outcomes),^47^ patients with co‐morbid SEA with CRSwNP (compared with those with SEA alone) were more likely to achieve minimum clinically significant 1‐year improvements in Asthma Control Test or ACQ‐6 (73.0% vs. 65.2%).^49^

**FIGURE 3:**
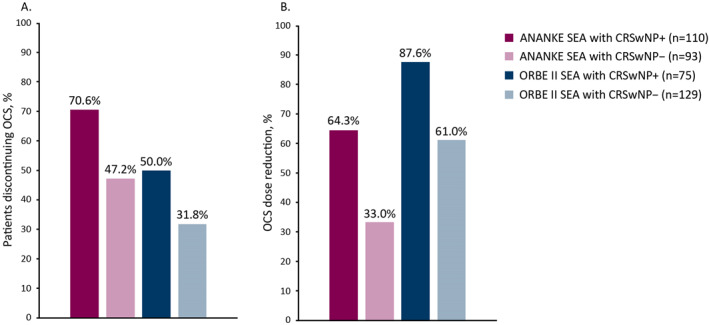
Changes in oral corticosteroid (OCS) use in ANANKE and ORBE, in terms of percent of (A) patients discontinuing OCS, and (B) OCS dose reduction.^46^, ^48^. OCS, oral corticosteroids.

Analyses of data from the XALOC‐1 component studies ANANKE and ORBE II have also produced interesting results in patients with co‐morbid disease. Both studies found that patients with co‐morbid SEA with CRSwNP were more likely to discontinue oral corticosteroids (OCS) following benralizumab treatment, compared with those with SEA alone (ANANKE, 70.6% vs. 47.2%; ORBE II 50.0% vs. 31.8%), and were more likely to achieve larger percentage reductions in mean daily OCS dose (ANANKE, 64.3% vs. 33%; ORBE II, 87.6% vs. 61.0%) (Figure 3).^46^, ^48^ Similarly, patients with co‐morbid SEA with CRSwNP in ANANKE were more likely to be exacerbation free, compared with those with SEA alone, following benralizumab therapy (89.5% vs. 71.9%).^46^ Patients with co‐morbid SEA with CRSwNP in ORBE II also experienced greater increases in FEV_1_ following benralizumab therapy compared with patients with SEA alone (mean [SD] improvement, 426 [420] mL vs. 277 [401] mL) and were more likely to have FEV_1_ ≥80% at the end of the 1‐year follow up period (52.2% vs. 43.2%).^48^ Furthermore, following benralizumab treatment, ORBE II patients with co‐morbid disease versus patients with SEA alone were less likely to have SEA‐related hospitalizations (96.7% vs. 88.6% reduction in rate from baseline) or emergency department visits (88.0% vs. 85.0% reduction in rate from baseline).^48^

### 2.5 Unmet needs: Research

While the data given above are compelling, it must be remembered that current evidence for the efficacy of benralizumab as a therapy for co‐morbid SEA with CRSwNP consists of (Table 1):

1. Two large‐scale retrospective real‐world studies.^44^, ^45^
2. Incidental data from SEA or CRSwNP focused studies, both RCTs and retrospective analyses.^8^, ^41^, ^43^, ^46^, ^47^, ^48^, ^49^, ^54^
3. Some smaller‐scale efforts.^42^, ^50^, ^51^, ^52^

Furthermore, it cannot be discounted that CRSwNP is acting as a biomarker for eosinophilic inflammation or more severe disease in these analyses, and that this is the reason that patients with comorbid disease demonstrate greater improvements with benralizumab treatment versus patients with SEA or CRSwNP alone.

Given the high HCRU and HRQoL burden common in patients with co‐morbid SEA with CRSwNP,^4^, ^5^, ^28^, ^29^, ^30^ there is an urgent need for further research concerning the efficacy of biologic therapies in this multimorbid phenotype. Taking into account the limitations of retrospective studies in terms of measuring and controlling outcome and potential confounding, these studies would ideally adopt prospective designs. There also remains a need to understand the importance of inflammatory cell heterogeneity in the etiology and management of eosinophilic‐associated inflammatory conditions.^55^, ^56^

The phase 3 ORCHID (Efficacy and Safety Study of Benralizumab in Patient With Eosinophilic Chronic Rhinosinusitis With Nasal Polyps; NCT04157335) study was a randomized, double‐blind, placebo‐controlled, parallel‐group, international, multicenter, phase 3 study, looking at the efficacy and safety of benralizumab in patients with CRSwNP and asthma.^57^ The co‐primary endpoints were change from baseline in endoscopic total nasal polyp score and change from baseline in mean nasal blockage score. Secondary endpoints included sense of smell, sinus opacification, SNOT‐22 score, time to endoscopic sinus surgery, time to corticosteroid use for CRSwNP, and nasal symptom scores. According to the top‐line results,^57^ the co‐primary endpoints were not met when comparing benralizumab versus placebo (complete primary data read‐out expected in 2025). It should be noted that although ORCHID included patients with asthma, it was not stipulated that patients had to have SEA. As such, there remains a lack of data from prospective RCTs focusing exclusively on the efficacy of biologic therapies such as benralizumab in patients with co‐morbid SEA with CRSwNP.

### 2.6 Unmet needs: Integrated management

Clinical evidence for the efficacy of benralizumab as a treatment for co‐morbid SEA with CRSwNP should ideally exist alongside a multidisciplinary framework for treating the condition.^17^ Reflecting the design of RCTs to date, current management approaches treat SEA and CRSwNP as separate entities, creating a risk for confused care approaches, duplication of work, and reduced patient welfare.^58^, ^59^ Very few studies have been published concerning integration of SEA with CRSwNP management,^60^ although experiences of specialists in other conditions suggest that multidisciplinary co‐ordination in asthma, allergy, and nasopharyngeal conditions holds potential to identify patients with co‐morbid disease and appropriately manage their underlying T2 inflammation.^9^, ^12^, ^13^, ^14^, ^15^, ^60^, ^61^

**FIGURE 4:**
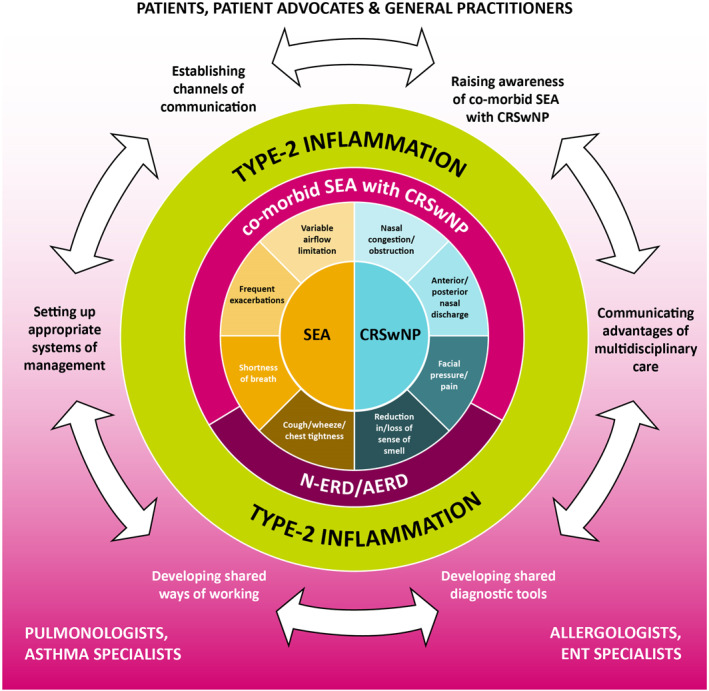
Proposed multidisciplinary framework for the management of co‐morbid SEA with CRSwNP. AERD, aspirin‐exacerbated respiratory disease; CRSwNP, chronic rhinosinusitis with nasal polyps; N‐ERD; NSAID‐exacerbated respiratory disease; NSAID, non‐steroidal anti‐inflammatory drug; SEA, severe eosinophilic asthma.

Multidisciplinary management is presently a topic of interest across a wide range of medical fields, especially those concerned with chronic conditions.^9^, ^61^ Various approaches have been proposed, ranging from co‐located services, and dedicated multidisciplinary teams, to the alignment of clinical goals and the implementation of integrated care pathways.^62^, ^63^, ^64^ Based on extant research, it is likely that necessary steps toward the development of a multidisciplinary care framework for co‐morbid SEA with CRSwNP include (see Figure 4):

‐ Raising clinical awareness of the SEA/CRSwNP overlap.^12^, ^13^
‐ Establishing and communicating the advantages of multidisciplinary SEA with CRSwNP care, in terms of HCRU and HRQoL.^11^
‐ Developing appropriate shared diagnostic tools for upper and lower airways (including the sense of smell).^12^, ^13^, ^61^
‐ Developing shared national and international treatment guidelines (i.e. European Position Paper on Rhinosinusitis and Nasal Polyps [EPOS], International Consensus Statement on Allergy and Rhinology [ICAR], Spanish Consensus on the Management of Chronic Rhinosinusitis With Nasal Polyps [POLINA]), lexicons, and ways of working.^13^, ^61^
‐ Encouraging communication between and shared education of clinicians involved in managing SEA or CRSwNP, including academic symposia and congresses.^13^, ^65^
‐ Setting up appropriate systems for referral, diagnosis, management, and follow‐up. These may include hospital‐based, multidisciplinary, working groups.^13^, ^61^
‐ Empowering patients with knowledge of the unified underlying mechanism and frequent co‐occurrence of SEA and CRSwNP.^17^

## 3 CONCLUSIONS

SEA and CRSwNP not only pose significant individual clinical burdens but also share a T2 inflammation‐based pathophysiology, leading to an elevated risk of co‐occurrence. Cases of co‐morbid SEA with CRSwNP often require complex clinical management and challenge the efficacy of traditional therapeutic approaches, which tend to address SEA and CRSwNP as individual diseases. Consequently, there is an urgent need to improve clinical recognition of this co‐morbid condition and develop comprehensive, multidisciplinary, frameworks and tools supporting integrated diagnosis and management. However, although the co‐morbidity that defines the condition increases the complexity of treatment, it appears to also present an opportunity for systemic therapy success. In this context, and despite a lack of studies explicitly designed for this purpose, benralizumab has shown efficacy in treating co‐morbid SEA with CRSwNP.

## AUTHOR CONTRIBUTIONS

**Joaquim Mullol**: Conceptualization; writing—review and editing; writing—original draft. **Maria D'Amato**: Conceptualization; writing—review and editing; writing—original draft. **Eugenio de Corso**: Conceptualization; writing—review and editing; writing—original draft. **Joseph K. Han**: Conceptualization; writing—review and editing; writing—original draft. **Jody Tversky**: Conceptualization; writing—review and editing; writing—original draft.

## CONFLICT OF INTEREST STATEMENT

Joaquim Mullol is or has previously been a member of advisory boards for and has received research grant projects and/or speaker fees from Almirall, AstraZeneca, GlaxoSmithKline, Lilly, Menarini, Merck Sharp and Dohme, Mitsubishi Tanabe Pharma, Noucor/Uriach Group, Novartis, Procter and Gamble, Regeneron Pharmaceuticals, Inc., Sanofi‐Aventis, UCB, and Viatris/MEDA. Eugenio de Corso has received lecture fees and has participated in advisory board meetings for GlaxoSmithKline, Novartis, Sanofi, AstraZeneca, and Firma. Joseph K Han is a research consultant for Genzyme, Sanofi Regeneron, GlaxoSmithKline, and AstraZeneca. Jody Tversky has received research funding from Regeneron, Sanofi, AstraZeneca, the American Thoracic Society, and the National Institutes of Health. Maria D'Amato does not have any disclosures of interest to declare.

## Data Availability

Research data are not shared.
